# Traditional Hands-On Puzzle Method versus Fully Digital Approach in Teaching Tooth Morphology: A Comparative Study of Educational Outcomes

**DOI:** 10.3390/dj12060162

**Published:** 2024-05-27

**Authors:** Amer Sehic, Qalbi Khan

**Affiliations:** Institute of Oral Biology, Faculty of Dentistry, University of Oslo, Blindern, P.O. Box 1052, 0316 Oslo, Norway; qalbi.khan@odont.uio.no

**Keywords:** dental anatomy, digital learning, e-learning, tooth identification, tooth morphology

## Abstract

Objective: Tooth morphology education is a critical component of dental curricula, providing a foundational understanding of the intricate structural anatomy of teeth. This study evaluates the learning outcomes in relation to tooth morphology of two student cohorts from different academic terms, comparing the traditional ‘tooth puzzle’ method to an alternative fully digital approach. Materials and Methods: Two groups of Master of Dentistry students were retrospectively analyzed. The control group (55 students) was taught via the ‘tooth puzzle’ method in 2021, while the experimental group (55 students) underwent a fully digital course in 2020 due to COVID-19 restrictions. Both groups completed a digital examination involving the identification of 40 teeth, presented as images and videos. Results: In the control group of 55 students, nearly half (49.1%) achieved faultless results, while 65.5% had at most two faults, and 74.5% had no more than four faults. The group had a total of 163 faults, averaging 3.0 per student, with only one student (1.8%) failing the test. In stark contrast, the experimental group had no students without faults, 9.1% had four or fewer faults, and a significant 61.8% made 10 or more faults, with 29.1% failing their first test attempt by exceeding 12 faults. Overall, the experimental group registered 582 faults, averaging 10.6 per student. Conclusions: The ‘tooth puzzle’ method, with its interactive and tactile elements, proved more effective in teaching tooth morphology than the digital-only approach. The increased number of faults and failed tests in the experimental group suggest that while digital tools offer meaningful support in learning tooth morphology, their main advantage is seen when coupled with traditional hands-on techniques, not unassisted and independently.

## 1. Introduction

The study of tooth morphology is essential in dental education, as it lays the groundwork for understanding the complex structural anatomy of teeth [1]. This knowledge is pertinent across all facets of dentistry. Educational strategies for imparting this knowledge vary, including traditional lectures, tooth carving exercises, examination of real and artificial teeth, analysis of dental illustrations, and the utilization of several different specialized E-learning supplements [2,3,4,5,6,7].

Didactic lectures still maintain a central position in the teaching of tooth morphology probably because of programs like PowerPoint, which enables making presentations in an easy and chronological way and offer new ways of visualizing videos, animations and images [8,9]. Teaching through the use of E-learning tools, which are independent and interactive, along with the implementation of virtual reality (VR) [10,11], has been reported in several studies but is understood to have an impact only as a valuable supplementary resource [6,12,13]. Hence, to develop a profound understanding of anatomical details, a practical course has been considered crucial, specifically in terms of dental anatomy carvings or the use of plastic teeth [14,15]. However, the biological features of teeth contain various anatomical variations that are hard to teach by using plastic teeth or through carving. A course that utilizes extracted teeth may, therefore, offer an invaluable educational advantage, given that issues concerning ethics and health are met [2,7,14].

In the more modern, flipped classroom model, the blending of digitized and practical courses has been described as advantageous for learning. Here, students are first introduced to key concepts through short, pre-recorded lectures. Second, they meet up for practical courses, preferably in groups, to discuss and develop the themes introduced online [16,17,18]. However, the flipped classroom model alone has recently been reported to not enable dental carving exercises, reflecting its limitations [19]. We, however, recently suggested that the most suitable teaching methodology for dental anatomy is the one that will be able to merge elements of the flipped classroom model with a practical course that applies the tooth identification puzzle method using extracted human teeth [20].

At the Institute of Oral Biology, Faculty of Dentistry, University of Oslo, the ‘tooth puzzle’ pedagogy is a hallmark of our curriculum, designed to impart an in-depth understanding of tooth morphology through direct interaction with real teeth, underscoring the importance of tactile and visual learning for a full grasp of dental anatomy. The ‘tooth puzzle’ instructional approach leverages the concrete interaction with real teeth to enhance tactile and visual learning, which is essential for a holistic grasp of dental anatomy [7]. Designed to deliver thorough comprehension in a streamlined timeframe, the course aims to be both enlightening and engaging. The primary task involves the accurate placement and identification of each tooth within a schematic dental chart, adhering to the FDI notation system [7].

A recent evaluation of this educational approach included a thorough post-course examination focused on assessing the students’ proficiency in tooth identification, underscoring the importance of a deep understanding of dental morphology. The course offers a condensed yet thorough exploration of the topic, aiming to be both engaging and rewarding. It begins with foundational lectures, followed by a 12 h practical session focused on the hands-on identification of a full set of extracted teeth, using the FDI World Dental Federation’s notation system. This approach is not only cost-effective but also enriches learning by engaging multiple senses, fostering a deep appreciation of morphological variations, and encouraging student–teacher interactions [7].

The course is currently taking place in the pre-clinical years. While its impact as a foundational dental course remains unquestionable, it should be stressed that the ongoing repetitions through short videos, as in a flipped classroom model, E-learning material and digital quizzes for periodic reassessment as students progress towards the end of their Master of Dentistry program is crucial, as a recent study undermines the clinical relevance [21].

The 2020 academic year presented unique challenges due to COVID-19, prompting adjustments to the delivery of our course to accommodate government-imposed restrictions. The adapted teaching strategy included a blend of 2D and 3D virtual tools, recorded lectures, and live online discussions to ensure continuous, interactive learning. This paper examines the outcomes of our established ‘tooth puzzle’ teaching method [7] in comparison to the improvised fully digital approach, assessing their effectiveness in a changed educational landscape.

## 2. Materials and Methods

**Figure 1:**
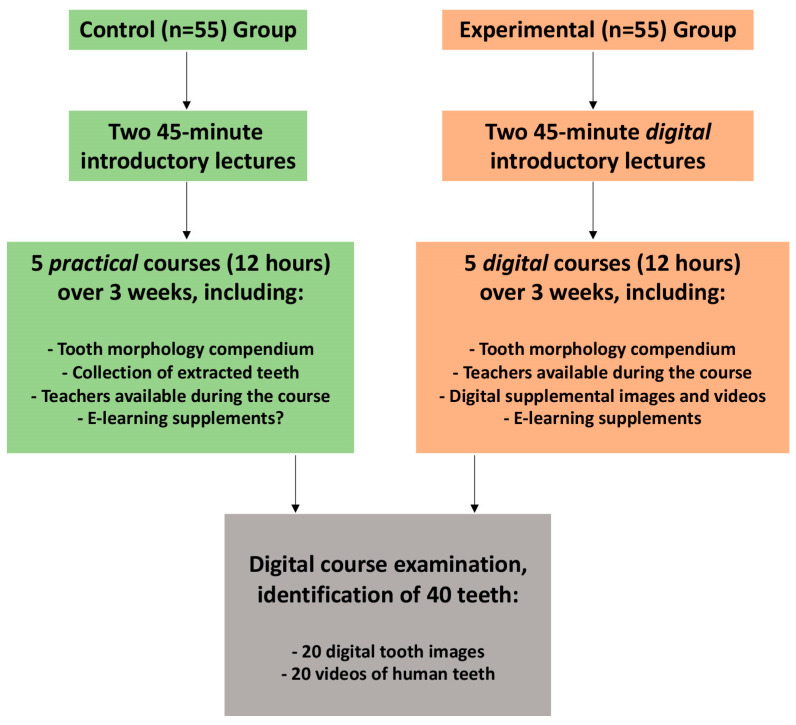
Study design and methodology: This flowchart outlines the structure of the study, comparing two distinct pedagogical approaches. The control group, consisting of 55 students, experienced the tactile ‘tooth puzzle’ method, including access to real teeth and extensive study materials. In contrast, the experimental group of 55 students underwent a digital learning path, complete with virtual lectures and a suite of online resources. Both groups had the compendium for reference, but the control group also had the benefit of practical, hands-on practice, while the experimental group had additional digital content to supplement their learning.

This retrospective study compares the outcomes of two Master of Dentistry student groups enrolled in a tooth morphology course across different academic terms, employing distinct teaching methodologies. The control group, comprising 55 students, participated in the established ‘tooth puzzle’ teaching method in 2021 [7]. Conversely, the experimental group, with 55 students, engaged in a fully digital course format in 2020, necessitated by COVID-19 pandemic restrictions (Figure 1). All of the students were informed about the study and were recruited as students of the Faculty of Dentistry at the University of Oslo during the second year of their Master of Dentistry program. The age and sociodemographic background are comparable due to the fact that the majority of the students are between 20 and 25 years old, and the sociodemographic differences in Norway are relatively small. However, females outnumbered males, comprising about 80% of the student group.

**Figure 2:**
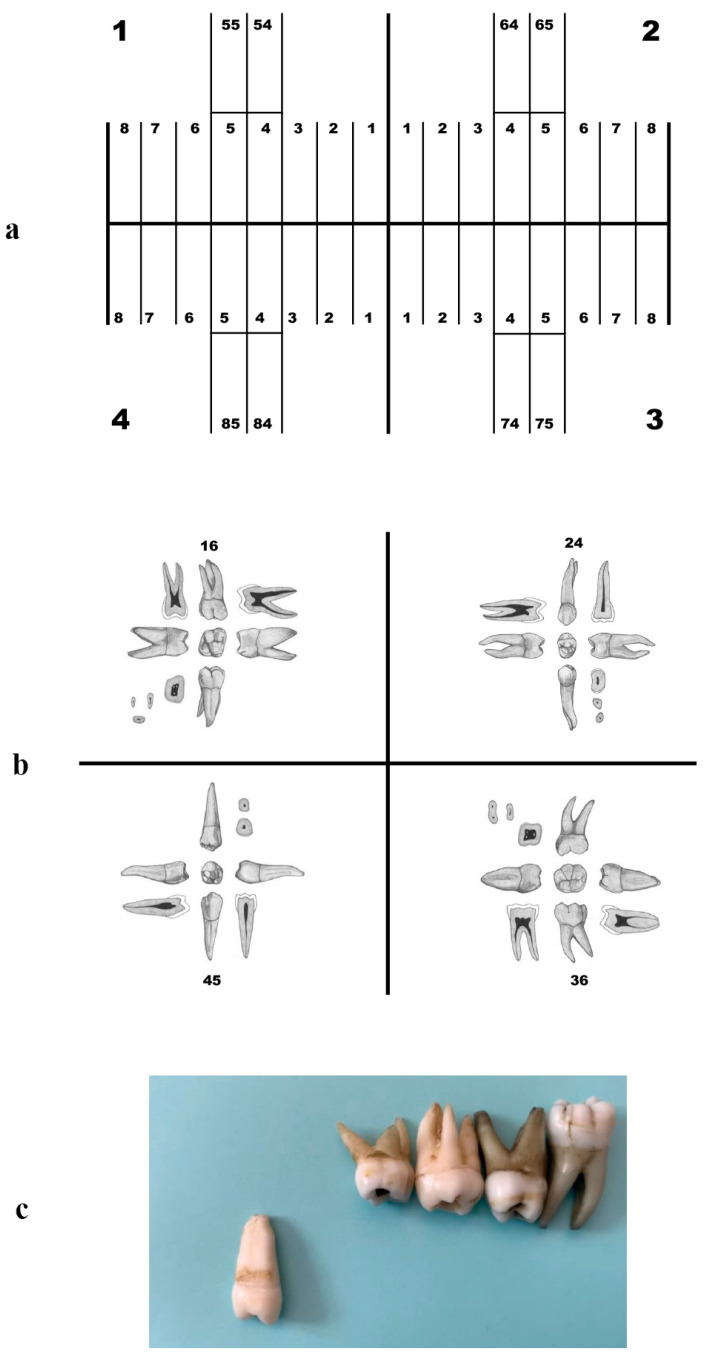
Study engagement and assessment format: The figure illustrates the hands-on and assessment components of the study. Students worked with 40-tooth sets to identify and arrange each tooth according to the FDI notation system (**a**). The course culminated in a digital examination comprising two modalities: static images (**b**) and dynamic video demonstrations (**c**), with students required to identify a total of 40 teeth across both formats.

The ‘tooth puzzle’ teaching method, detailed previously in the literature [7], involves a structured educational experience for the control group. Initially, students attended two 45 min lectures, providing an overview of the course content. Subsequently, a comprehensive 12 h practical segment on tooth identification was distributed over a three-week span. During this practical phase, students worked with sets of extracted teeth, each comprising the full array of 32 permanent teeth and 8 deciduous molars. These teeth of undisclosed origin, either sent to the Faculty of Dentistry by affiliated dental offices or collected from the student clinic, were sanctioned for educational use. No additional hygienic instructions were given to the students than to follow general hygienic protocols. The teeth have been preserved in 70% alcohol for several years in glass jars and are thoroughly dried out to ensure the elimination of any organic matter before use. Organized into bags, these 40-tooth sets are allocated to students either individually or in small groups based on the availability of materials and student preference. The task is to correctly identify and position each tooth on a schematic dentition diagram using the FDI notation system (Figure 2a). Students have access to approximately 30 sets of teeth, a tooth morphology compendium enriched with detailed drawings, descriptions, and identification tables, as well as the opportunity for additional practice on faculty premises. While no additional digital resources such as videos or images are provided by the educators, the students in this group were granted the autonomy to utilize various e-learning supplements at their discretion during their personal study time.

In contrast, the experimental group from 2020 commenced their learning with two recorded 45 min digital lectures, accessible throughout the course duration. In addition to the same compendium provided to the control group, these students completed a 12 h digital course over three weeks. The course was designed to allow instructors to present material and monitor student engagement and progress. Supplementary digital resources, including videos, images, and external e-learning tools, such as digital atlases and applications, were incorporated. Prior to each of the five sessions, students were required to watch a dental anatomy lecture, review the selected parts of the compendium, and complete a pre-quiz designed to reinforce preparation, motivate active participation, and facilitate discussion on key concepts. The online sessions involved collaborative in-class activities, with students divided into groups of 6–8, focusing on specific topics.

Both groups concluded their coursework with a digital examination, which entailed the identification of 40 teeth—half presented as images and the remaining as video demonstrations (Figure 2b,c). In the video demonstrations, each tooth was methodically presented from multiple perspectives. The sequence commenced with the occlusal/incisal view and was followed by the facial, lingual, mesial, and distal aspects to provide a comprehensive visual assessment. During this evaluation, students were not permitted any reference materials. A maximum of 12 incorrectly identified teeth was deemed the threshold for passing the examination.

**Figure 3:**
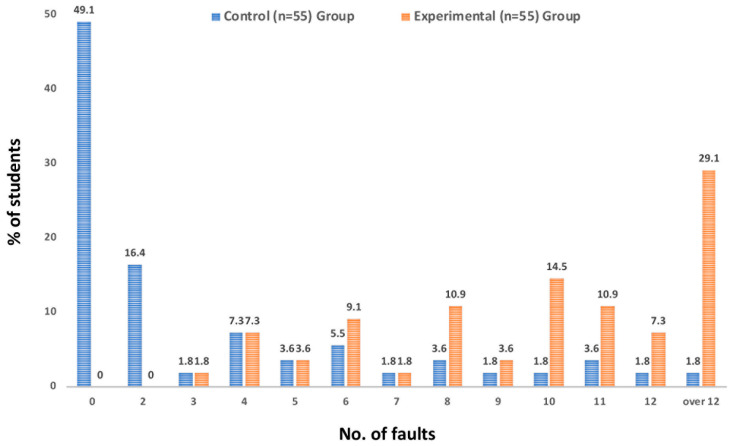
Consolidated outcomes of the tooth identification examination: This figure presents a comprehensive breakdown of the final test results from the tooth morphology course, illustrating the comparative performance of the control and experimental groups. It quantifies the percentages of students within each group in relation to the number of faults they incurred during the identification test. The figure serves to visually convey the distribution of fault occurrences across both cohorts, offering insight into the efficacy of each teaching method.

The number of incorrectly identified teeth was meticulously documented. The aggregated data on the students’ performance were recorded as the number of faults related to both the control and experimental groups (delineated in Table 1). The percentages of students within each group in relation to the number of faults were quantified in a Windows Excel Worksheet and are represented as a histogram in Figure 3. The type of fault was also registered, and the percentage was calculated (delineated in Table 2).

## 3. Results

In the control group, out of 55 students, 27 (49.1%) exhibited perfect precision with no faults. A total of 36 students (65.5%) had no or two faults, and 41 students (74.5%) had four or fewer faults. Overall, the control group accumulated 163 faults, averaging 3.0 faults per student. Notably, only one student (1.8%) failed the assessment by committing more than 12 faults, specifically 22 faults.

In contrast, the experimental group’s performance was markedly different. Of its 55 students, none (0%) managed to have no or two faults, and only 5 students (9.1%) kept their faults to four or fewer. On the higher end of the fault spectrum, 34 students (61.8%) incurred 10 or more faults, and 16 students (29.1%) did not pass the test on their first try due to having over 12 faults. In total, the experimental group made 582 faults, which translates to an average of 10.6 faults per student. The highest number of faults recorded for an individual in the experimental group was 34.

When re-tested approximately a week later after receiving additional exercises and instruction, the one student from the control group who had failed passed the assessment. From the experimental group, only 7 out of the 16 who did not pass initially were able to succeed on their second attempt. After another week of intensified exercises and further instruction, the remainder of the students from the experimental group also passed.

In total, 7.4% (163 out of 2200) of all positioned teeth were incorrectly placed by the control group, whereas the experimental group had a significantly higher misplacement rate of 26.5% (582 out of 2200). Analysis of the control group’s data revealed that the most misplaced teeth were the central mandibular incisors, followed by the second maxillary premolars, the first mandibular premolars, the second mandibular incisors, and the maxillary third molars. Collectively, these five types of misplacements accounted for 50.9% (83 of 163) of the control group’s total faults (Table 2). Conversely, the experimental group’s faults did not follow a discernible pattern concerning the type of tooth misplacement; the faults were dispersed throughout the various positions in the dentition. When assessing the same five most misplaced teeth as in the control group, these accounted for only 25.8% (150 of 582) of the experimental group’s faults (Table 2).

## 4. Discussion

The adoption of a ‘tooth identification puzzle’ pedagogy in teaching tooth morphology presents a myriad of advantages for both the educators and the students, as discerned through our academic experience [7]. The most striking finding from this study is that the well-established and effective ‘tooth puzzle’ method, when adapted to a fully digital format that lacks hands-on interaction with real teeth, leads to markedly reduced proficiency in tooth morphology. This decline in knowledge retention was evident upon course completion and was objectively measured through structured identification tests.

The present retrospective analysis evaluates the performance of two cohorts of Master of Dentistry students in the tooth morphology course, each subjected to different teaching strategies during separate academic years. The control group of 55 students was taught through the traditional ‘tooth puzzle’ method, while the experimental group, also consisting of 55 students, received a fully digitalized curriculum in response to the constraints imposed by the COVID-19 pandemic (Figure 1). Upon completion of the course, both groups were assessed through a digital examination that required the identification of 40 teeth, with 20 displayed as still images and the remaining 20 presented through video demonstrations.

In the early phases of the course, both the control and experimental groups progressed at a measured pace to ensure students developed a solid grasp of the essential principles underlying tooth structure and familiarized themselves with the specific dental terminology. While both groups found the initial stages of the course challenging, the control group demonstrated a more pronounced improvement following this phase. Surprisingly, despite the digital group receiving more focused teacher attention for closer monitoring of their progress, their advancement did not match that of the control group. The digital course was meticulously crafted to permit instructors to present content interactively and oversee student engagement and progress. This included the integration of various digital resources such as videos, images, and external e-learning platforms, including digital atlases and applications. Each of the five sessions commenced with a dental anatomy video lecture, followed by a review of relevant sections from the compendium and a preparatory quiz, all designed to enhance readiness, encourage participation, and guide discussions on crucial concepts. In these online sessions, students engaged in cooperative tasks, grouped into teams of 6–8 to tackle specific subjects. In contrast, the control group, after initial lectures, primarily engaged with real teeth, either individually or in smaller groups. This hands-on approach proved to be quite effective. It is postulated that incorporating a gamified element into the curriculum may significantly bolster the learning process, rendering it a more pleasurable experience that also promotes significant skill development among the students.

This study brought to light significant challenges within the educational process. The control group, subjected to a digital test, mostly misplaced the central mandibular incisors, second maxillary premolars, first mandibular premolars, second mandibular incisors, and maxillary third molars. This outcome is intriguing when compared to the prior groups evaluated [7], which underwent a practical test; despite the change in assessment format, the control group’s performance on the digital test was nearly as proficient as the previous groups’ on the practical test. This consistency is evident in the pattern of the most common misplacements, with the notable exception of the maxillary third molars in this group compared to the mandibular first molars previously [7]. In contrast, the experimental group, which received solely digital instruction, did not exhibit a consistent pattern in tooth misplacement; the faults were randomly distributed across different dental positions. For the same five teeth most misplaced by the control group, the experimental group’s faults for these teeth constituted only 25.8% of their total misplacements. This suggests that the experimental group’s learning experience, which was exclusively digital, did not facilitate dental anatomy comprehension as effectively as the control group, as evidenced by the varied and less structured faults observed across different tooth categories and jaw regions. The findings prompt a secondary conclusion that engaging in tactile exploration, particularly of nuanced dental characteristics like root furrows, is instrumental in fostering a more holistic and in-depth educational experience. This hands-on approach appears to be crucial in developing a nuanced appreciation and understanding of dental anatomy, underscoring the importance of sensory interaction in the mastery of morphological details.

In another study, an online tooth morphology course was developed as an adaptive measure to COVID-19 restrictions [22]. This course included an integrated 3D learning tool designed to provide immediate feedback for feature identification, a feature that might contend with the use of real extracted teeth. The performance of the 2021 cohort on examinations aligned with the averages of the 2016 to 2019 cohorts. The authors recommended further research to determine the efficacy of translating virtual 3D learning experiences to the identification of physical teeth and the potential of such technologies to replace traditional in-person instruction. While the current study did not explore this translation to physical identification, it would have been insightful to evaluate whether the experimental group of this study could apply their digital learning to real teeth. Nonetheless, the breadth of fundamental knowledge gaps indicated by their performance on digital assessments suggests that such an exercise might not significantly alter the overarching conclusions about their acquired knowledge. The implementation of software-assisted teaching in a dental morphology course has previously been demonstrated to enhance student learning outcomes when used as a complementary resource rather than as the sole educational tool [23].

A previous investigation revealed that at-home waxing exercises, supported using step-by-step imagery and instructional videos within the 3D Tooth Atlas, yielded promising outcomes [24]. However, students expressed a marked preference for direct feedback from faculty, a component missing in the at-home setting. While students successfully grasped the didactic elements of tooth morphology via webinars augmented by the 3D Tooth Atlas, the majority showed a predilection for traditional in-person classes that allowed for direct engagement with faculty and peers. This inclination underscores the inherent human need for interpersonal interaction. Consistent with this finding, the long-standing experience with the ‘tooth puzzle’ course, as described here, corroborates the value of a cooperative learning environment. Such an environment establishes a foundational platform for fostering positive and dynamic interactions between students and faculty, which is critical to creating an enriching educational setting. This, in turn, contributes to both academic success and the overall well-being of the students.

The current study has its limitations, specifically in the use of only one experimental group. Additionally, only two observers/teachers participated in this study. Furthermore, due to the pandemic, personal factors such as disease, death in the family, psychological issues, and loneliness may have influenced the outcome scores of the test. In addition, gender differences were not accounted for due to the fact that the females outnumbered the males, which would have yielded too little data on the male part. In the future, more emphasis should be placed on investigating for gender differences, along with the contributing factors of an outbreak.

## 5. Conclusions

A thorough education in tooth anatomy equips students with essential skills for addressing practical dental challenges. Multisensory engagement and interactive learning are pivotal in fostering a comprehensive educational experience [14,15]. This study posits that there are limits to digital learning. It may provide a robust alternative for students who thrive in self-directed learning environments or as a complement to hands-on experience with real teeth. However, for teaching dental anatomy as described in this study (where the course imparts a practical knowledge of the variability in tooth morphology as students explore and learn from an array of tooth sets), its success is rooted in an engaging learning milieu that encompasses direct observation and hands-on interaction with real human extracted teeth, complemented by peer collaboration, instructor guidance, and detailed study materials. It is also noted that there is variability in how learners interpret 2D information into 3D understanding, with some finding the translation intuitive and others facing difficulties.

## Figures and Tables

**Table 1:** Student performance analysis: The table compiles the performance metrics of students from both the control and experimental groups. The counts and corresponding percentages of students are displayed, categorized by the number of faults they incurred. Additionally, the table provides the total sum of faults for each group, offering insight into overall accuracy and precision within the course assessments.

	Control (n = 55) Group		Experimental (n = 55) Group	
No. of Faults	No. of Students (%)	Total No. of Faults	No. of Students (%)	Total No. of Faults
0	27 (49.1%)	0	0 (0%)	0
1	0 (0%)	0	0 (0%)	0
2	9 (16.4%)	18	0 (0%)	0
3	1 (1.8%)	3	1 (1.8%)	3
4	4 (7.3%)	16	4 (7.3%)	16
5	2 (3.6%)	10	2 (3.6)	10
6	3 (5.5%)	18	5 (9.1%)	30
7	1 (1.8%)	7	1 (1.8%)	7
8	2 (3.6%)	16	6 (10.9%)	48
9	1 (1.8%)	9	2 (3.6%)	18
10	1 (1.8%)	10	8 (14.5%)	80
11	2 (3.6%)	22	6 (10.9%)	66
12	1 (1.8%)	12	4 (7.3%)	48
over 12	1 (1.8%)	22	16 (29.1%)	256
	55 (100%)	163	55 (100%)	582

**Table 2:** Predominant categories of tooth misplacements. This table enumerates the primary categories of tooth misplacements identified in the control and experimental groups. It quantifies the total faults recorded for each group and further breaks down the frequency and percentage of each fault type.

	Control Group (n = 163)	Experimental Group (n = 582)
Type of Fault	No. of Faults (%)	No. of Faults (%)
Central mandibular incisors	21 (12.9%)	23 (3.9%)
Second maxillary premolars	20 (12.5%)	31 (5.3%)
First mandibular premolars	17 (10.5%)	25 (4.3%)
Second mandibular incisors	13 (8.0%)	42 (7.2%)
Maxillary third molars	12 (7.4%)	29 (5.0%)

## Data Availability

The original contributions presented in the study are included in the article, further inquiries can be directed to the corresponding author.
